# Péritonites infectieuses en dialyse péritonéale continue ambulatoire au CHU de Rabat: profil bactériologique sur trois ans

**Published:** 2012-03-08

**Authors:** Zineb Lioussfi, Hakima Rhou, Fatima Ezzaitouni, Naima Ouzeddoun, Rabea Bayahia, Loubna Benamar

**Affiliations:** 1 Service de Néphrologie – Dialyse – Transplantation rénale, Hôpital Ibn Sina, Rabat, Maroc

**Keywords:** Dialyse péritonéale continue ambulatoire, Microbiologie, Péritonite infectieuse, Gram positifs, Gram négatifs

## Abstract

**Introduction:**

La péritonite infectieuse (PI) est une des complications les plus sévères et les plus fréquentes de la dialyse péritonéale (DP). But: Déterminer le taux des PI et les germes en causes, et évaluer l’efficacité des protocoles thérapeutiques entrepris chez les patients traités par DP au CHU de Rabat.

**Méthodes:**

Etude rétrospective effectuée en Septembre 2009 chez tous les patients traités par DP continue ambulatoire (DPCA) au CHU de Rabat depuis l’ouverture de l’unité de DP en Juillet 2006. Ont été inclus dans cette étude, tous les patients ayant fait une péritonite. Pour tous nos patients, nous avons relevé les données cliniques, biologiques et bactériologiques. Nous avons également recherché les causes des péritonites, le délai de survenue par rapport au début de la dialyse, et la durée moyenne de formation des patients.

**Résultats:**

Au cours de la période de l’étude, 28 épisodes de PI sont survenus chez 19 patients dont la moyenne d’âge est de 46±16 (19-78) ans, avec une prédominance masculine (12 hommes/ 7 femmes). Le taux des PI dans notre unité de DP est de 21.07 mois-patients calculé par le RDPLF. Leur délai de survenue par rapport au début de la dialyse au centre est de 7.9 ±8 (1-29) mois. Lors de ces PI, les bactéries à Gram négatif (BGN) ont été retrouvées dans 55% des cas, contre uniquement 45% pour les Gram positifs.

**Conclusion:**

La PI est une complication grave et redoutable de la DP. Le taux de PI dans notre centre de DPCA est de 21m-p ce qui correspond au taux acceptable définie par les sociétés internationales. Les germes les plus responsables des PI dans notre centre sont les BGN et la contamination semble être manu-portée se faisant essentiellement à partir de la flore environnementale et cutanée.

## Introduction

La dialyse péritonéale (DP) est une méthode d’épuration extra-rénale qui peut être proposée en 1^ère^ intention pour la prise en charge de l’insuffisance rénale chronique terminale. Les patients mis sous DP peuvent être confrontés à différentes complications d’ordre mécanique ou infectieux. La péritonite infectieuse (PI) constitue une des complications les plus sévères [1] et les plus fréquentes de la DP: environ deux tiers des patients développent une péritonite au cours de la première année de dialyse [2].

Malgré la diminution du taux de ces infections avec l’introduction du système double poche et l’amélioration des systèmes de connexion [3], la péritonite demeure le tendon d’Achille de la dialyse péritonéale (DP) [4] et la cause la plus importante de sortie de la technique vers l’hémodialyse [5]. En plus, les infections réfractaires au traitement et/ ou à répétition peuvent altérer la membrane péritonéale [6] avec majoration des pertes protéiques et augmentation de l’absorption du glucose [1], source de dénutrition et de surcharge hydro-sodée.

Vu les effets néfastes de ces infections sur la survie de la technique ainsi que sur la mortalité des patients mis sous DP, plusieurs pays ont tenté de mettre au point des protocoles de prise en charge, adaptés à leur écologie bactérienne et aux moyens disponibles [7].

Au Maroc, et jusqu’à l’heure actuelle, il n’a pas été mis en place de protocole particulier adapté aux germes les plus fréquemment isolés, et leurs sensibilités aux différents antibiotiques disponibles. Ainsi, nous avons suivi les recommandations de la Société Internationale de DP (ISPD) qui ont été publiées depuis 1983, et révisées à plusieurs reprises en 1989, 1993, 1996, 2000 et 2005 [7,8].

Le but de notre étude est de déterminer le taux des PI survenant chez les patients traités par dialyse péritonéale continue ambulatoire (DPCA) au CHU de Rabat, et de préciser l’écologie bactérienne dans notre centre, ainsi que l’efficacité des protocoles thérapeutiques entrepris.

## Méthodes

La dialyse péritonéale a été initiée dans notre CHU en Juillet 2006. Sur une période de 38 mois, depuis l’initiation de la technique, 38 patients ont été suivis dans notre centre, tous traités par DPCA. L’âge moyen des patients est de 47±17.8 (10-78 ans) avec un sex-ratio de 1.6. Parmi ces patients 21% sont diabétiques.

Il s’agit d’une étude rétrospective effectuée dans notre centre en Septembre 2009. Ont été inclus, tous les patients suivis en DP dans notre centre. Pour les patients ayant fait une PI, nous avons analysé les données démographiques (l’âge, le sexe, la durée de dialyse, la néphropathie initiale et le niveau socio-économique), et les signes cliniques lors des péritonites (douleur abdominale, fièvre, turbidité de l’effluent et diarrhée). Nous avons relevé des données biologiques (taux de globules blancs et CRP), et bactériologiques (nombre de leucocytes et de PNN dans le dialysat drainé et germes identifiés). Nous avons également recherché le portage nasal du staphylocoque doré concomitamment à la péritonite, et la présence d’une infection du site d’émergence. Le délai de formation des patients et le délai de la péritonite par rapport à la pose du cathéter et par rapport au début des échanges ont également été relevés.

Les prélèvements bactériologiques du dialysat ont été effectués après l’apparition de signes cliniques tels que la turbidité du dialysat drainé, associé ou non à une fièvre ou une douleur abdominale. Les prélèvements sont faits sur la poche trouble ramenée par le patient, et sur la poche de dialysat drainé à l’hôpital. Un 1er prélèvement est effectué à l’aide d’une seringue stérile après agitation du contenu de la poche pour avoir un comptage cellulaire. Après avoir laissé la poche suspendue pendant 20 min, d’autres prélèvements sont effectués, ils comprennent d’une part un examen microscopique direct après coloration de Gram d’un frottis réalisé à l’aide d’une cyto-centrifugeuse et d’autre part une culture en milieux liquides par ensemencement de 5 ml de liquide péritonéal en flacon d’hémoculture aérobie et anaérobie. Une étude mycologique ainsi que la recherche de *Mycobactérium tuberculosis* sont systématiquement réalisées. La sensibilité aux antibiotiques de toutes les souches isolées a été systématiquement étudiée. En complément de l’analyse du liquide péritonéal, une recherche systématique et régulière du staphylocoque au niveau du nez et du site d’émergence a été effectuée de même qu’un examen bactériologique des urines et des hémocultures afin de rechercher une éventuelle source d’infection.

Le diagnostic de péritonite infectieuse est posé après confrontation des résultats du laboratoire de bactériologie et des données cliniques. Ainsi le diagnostic de péritonite est retenu, si au moins deux des trois critères suivants sont réunis: 1) douleurs abdominales ou liquide péritonéal trouble, 2) nombre de leucocytes supérieur à 100 par mm^3^ dans le liquide de drainage avec un taux de neutrophiles supérieurs à 50% 3) culture bactériologique et/ou examen microscopique positif.

Les péritonites ont été traitées selon les recommandations de la Société Internationale de Dialyse Péritonéale (ISPD) par antibiothérapie empirique visant à la fois les germes Gram positifs et Gram négatifs. Nous avons ainsi administré une céphalosporine de troisième génération (1g par jour en intra péritonéal le soir) associée à une dose de vancomycine (1g en perfusion intraveineuse à renouveler après 5 à 7 jours selon le taux résiduel de vancomycine). Ce protocole a été modifié selon les résultats de la culture et de l’antibiogramme.

Nous avons adopté la terminologie de L’ISPD pour définir la nature et l’évolution des épisodes de PI survenues chez nos patients. Ainsi, l’amélioration clinique a été définie par la résolution des signes cliniques, l’éclaircissement du liquide de drainage et la diminution des leucocytes du dialysat drainé et l’efficacité thérapeutique est constatée devant la résolution complète de l’épisode sous traitement antibiotique seul sans recours à l’ablation du cathéter ni rechute ou récidive [8] (Tableau 1).

**Tableau 1:** Terminologie des épisodes infectieux selon la Société Internationale de Dialyse Péritonéale

Infection	Episode	Date de survenue	Germe isolé
Récurrente	Nouveau	≤ 4 semaines	Différent
Récidivante	Nouveau	≤ 4 semaines	Identique ou stérile
Répétée	Nouveau	> 4 semaines	Identique
Réfractaire	Le même	> 5 jours par un traitement adapté	Identique
Liée au cathéter	Infection du cathéter	Dans les 2 mois précédant la péritonite	Identique

## Résultats

Au cours de la période de l’étude, 28 épisodes de PI ont été diagnostiqués, survenus chez 19 patients dont la moyenne d’âge est de 46±16 (19-78) ans, avec une prédominance masculine (12 hommes/ 7 femmes). Soixante-dix pour cent de ces patients ont un niveau d’étude moyen ou bas (Tableau 2).

**Tableau 2:** Caractéristiques cliniques des patients et délai de survenue des péritonites infectieuses

Caractéristiques	Résultats
Nombre de patients	19
Sex Ratio (H/F)	1.7 (12/7)
Age moyen (ans)	46±16 (19-78)
Durée de dialyse (mois)	20±7 (6-29)
BMI kg/ m^2^	22,8±4.5
Surface corporelle m^2^	1,65± 0,2
Néphropathie initiale: n (%)	
Diabète	3 (16.6)
GNC	3 (16.6)
Indéterminée	6 (31.5)
Score de Charlson	4 ±2
Délai des péritonites par rapport au début de la dialyse (mois)	7.9±8 (1-29)

Depuis l’initiation de la technique dans notre CHU en juillet 2006 jusqu’à Septembre 2009, le taux de péritonites dans notre centre calculé par le RDPLF est de 21.07 mois-patients. Environ la moitié de nos patients en DP (52%) n’ont présenté aucun épisode infectieux durant la période de l’étude. Sur les 47% restants, un épisode infectieux par patients est survenu chez 34% des cas, deux épisodes par patients chez 8% des cas, et trois péritonites chez 5% cas.

Le délai de survenue des PI par rapport au début de la dialyse au centre est de 7.9 ±8 (1-29) mois. La durée moyenne de formation des patients est de 6±3 jours.

Lors de ces PI, le signe clinique prédominant est la turbidité de l’effluent notée chez tous nos patients. Les douleurs abdominales ont été rapportées chez 96% des cas, et l’hyperthermie chez seulement 40% des cas. Sur le plan bactériologique, tous les patients avaient un taux de leucocytes dans l’effluent supérieur à 100/mm^3^, avec un taux moyen de globules blancs dans l’effluent de 6180±700/mm^3^.

Ces PI étaient secondaires à une infection du site d’émergence (ISE) avec isolement du même germe à l’écouvillonnage du site d’émergence dans 4 cas. Les autres causes identifiées sont une faute d’asepsie de la part des patients ou dès le retour du bloc opératoire suite à l’insertion du cathéter de DP (8 cas), l’iatrogénie suite à des erreurs faites lors de la réalisation du Test d’équilibration péritonéale (2 cas), et une diarrhée non traitée (4 cas). La PI était accidentelle dans un cas suite à une déconnexion inopinée du circuit, l’antibiothérapie préventive n’a pas été administrée chez ce patient à cause d’un retard de consultation après cet incident. L’étiologie des PI est restée sans cause évidente dans le reste des cas. La recherche du portage nasal de staphylocoque concomitamment aux péritonites était négative chez tous les patients de même que les ECBU. Les hémocultures étaient négatives ne permettant pas d’identifier de bactériémie associée.

Ces infections étaient monomicrobiennes dans 60.7% des cas, et polymicrobiennes dans 10.7% des cas. Huit cultures sont revenues négatives. La preuve bactériologique a été apportée dans 20 cas avec isolement d’un germe par culture. Seuls 5 examens microscopiques sont revenus positifs et ont mis en évidence des bacilles Gram négatifs. Parmi les espèces isolées dans notre centre, 55% étaient des bactéries à Gram négatif et 45% des bactéries à Gram positif. Tous les prélèvements mycologiques sont revenus négatifs. Les bactéries anaérobies n’ont jamais été isolées de même que le *Mycobactérium tuberculosis*.

Les espèces isolées lors des PI à Gram positif sont un staphylococcus coagulase négatif (SCN) (4 cas), un *Staphylococcus auréus* (SA) (3 cas), et un streptococcus non groupable (SNG) (2 cas). Les péritonites à *Pseudomonas aerugenusa* étaient les plus fréquentes parmi les infections à Gram négatif (5 cas). Le *Klebsiella oxytoca* a été isolé lors de 2 épisodes différents survenant chez le même patient. L’Acinetobacter, L’*Escherichia coli*, l’*Enterobacter*, le *klebsiella pneumoniae* et le *Citrobacter freundii* ont été isolés chacun dans un seul cas.

La sensibilité aux antibiotiques (ATB) des différentes souches isolées a été testée systématiquement. Ainsi, parmi les cocci Gram positif un seul germe (11%) était résistant à la méthicilline et aucune résistance à la vancomycine n’a été retrouvée. Deux bacilles Gram négatifs (16.6%) étaient résistants aux quinolones et un seul *Pseudomonas aerugenusa* (8.3%) résistant à la ceftazidime (Tableau 3).

**Tableau 3:** Sensibilité aux ATB des germes isolés

	Espèce isolée	Examen direct+/ culture+	Sensibilité aux ATB
Cocci Gram Positive	*Staph aureus*	1/3	3 méthi-sensibles
	*Staph coagulase* négative	0/4	1 méti-résistant
	Staph non groupable	0/2	2 Pénicilline sensibles
	*Pseudomonas aerogenusa*	2/5	1 résistant à ceftazidime2 résistants à cefotaxime
Bacille Gram Négative	*Enterobacter*	0/1	Résistant à ceftriaxone
	*Klebsiella oxytoca*	1/2	1 résistant à ceftriaxone
	*Acinetobacter*	1/1	Sensibles aux C3G
	*Citrobacter freundeii*	0/1	Sensibles aux C3G
	*Escherichia coli*	0/1	Sensibles aux C3G

Tous nos patients ont reçu une dose de vancomycine renouvelable selon les résultats des dosages de la vancocynémie, associé à 1 gramme par jour de ceftazidime en intra-péritonéal. Une dose d’amikacine ou une quinolone étaient associées dans certains cas devant des signes de gravité cliniques. Ce traitement initié avant même d’avoir isolé un germe aux différents prélèvements effectués, est aussitôt réajusté selon les résultats de l’antibiogramme.

Les patients ayant fait une PI à Pseudomonas ou à SA ont bénéficié du protocole Urokinase. Celui-ci consiste en l’injection d’un fibrinolytique dans la lumière du cathéter, afin de lutter contre la formation du biofilm, résultat de la sécrétion de glycoprotéines par ces bactéries. Le drainage de la cavité péritonéale se fait dans les 4 heures qui suivent.

Le traitement des PI dans notre centre se fait en ambulatoire. Seuls trois patients ont nécessité une hospitalisation devant la gravité du tableau clinique suite à un retard de consultation après apparition des signes cliniques dans 2 cas, et vue l’aggravation de la symptomatologie dans un cas de PI à *Citrobacter Freundii*.

L’évolution a été marquée par l’amélioration des signes cliniques et l’éclaircissement de l’effluent chez 89% des patients. Trois patients ont présenté une infection réfractaire au traitement usuel dont une infection à Pseudomonas résistant à la ceftazidime ayant nécessité un traitement par Tienam, une 2^ème^ à *Citrobacter freundii*, et la 3^ème^ à culture négative. Vue la non amélioration clinique et biologique après 5 jours, l’ablation du cathéter s’est imposée chez les trois patients qui sont sortis de la technique et ont été mis en hémodialyse.

Trois récidives infectieuses (16%) ont été constatées et aucune rechute ni décès suite à une PI ne sont survenus.

## Discussion

En DPCA, le taux de PI acceptable pour une unité de DP est de 1 épisode tous les 18 mois- patient (m-p) selon l’ISPD [8] ou de 1 épisode tous les 30.6 m-p selon le RDPLF [9]. Notre taux de PI après trois ans d’expérience est de 21.07 m-p. Ce taux satisfaisant qui répond aux normes de la SIDP, est meilleur que celui enregistré en Ecosse [7] (1épisode/ 19.2m-p), et aux Royaume Uni [10] (14.7m-p). Il est cependant inférieur au taux de PI en Tunisie [11] (1/24 m-p), en France [12] (30 m-p) et à Hong Kong [13] (1/36 jusqu’à 45m-p après introduction des systèmes double poche).

L’ISPD [14] et l’Association Renal aux Royaumes Unis [15] exigent un taux de cultures négatives inférieur à 10% dans les centres de DP. Dans notre unité, la preuve bactériologique a été rapportée dans 59% des cas avec isolement d’un germe par examen direct et/ou par culture. Les 41% de cultures négatives pourraient être expliqué par notre manque d’expérience lors de l’initiation de la technique dans notre centre concernant la méthode de prélèvement bactériologique, et le mode d’acheminement au laboratoire. Plus de la moitié de nos péritonites sont dues à des germes Gram négatifs (55%), notamment *Pseudomonas aerugenusa* (45%), et *Klebsiella oxytoca* (18%). La même tendance à l’augmentation des infections à Gram négatif a été notée en Inde [16] (60%), à Hong Kong [13], et lors d’une étude réalisée en France s’étalant sur dix ans (de 1991 à 2001) [12].

Les bactéries à Gram + ont été isolées dans 45% des cas, avec une prédominance du SCN (4cas), suivi du SA (3cas). Un taux semblable (44%) a été noté par Gadola en Uruguay [17]. Laurain quant à lui avait rapporté une prédominance des Gram + dans un centre en France avec un taux allant jusqu’à 68% [12].

Bien que moins nombreux dans notre série, les SA ont un intérêt clinique important car comme l’a bien précisé Peacock et al. [18] les péritonites à SA sont souvent plus sévères que celles à SCN. Elles peuvent entraîner des complications abdominales et conduire à un retrait anticipé du cathéter ainsi qu’à un passage plus rapide en hémodialyse.

Une péritonite polymicrobienne est survenue dans 10.7% des cas. Ce chiffre rejoint celui de e Szeto et al [19] qui a rapporté 11% de cultures poly-microbiennes et Kim et al [20] qui en a noté 8%.

L’étude de la sensibilité des germes aux antibiotiques, n’a pas permis de mettre en évidence une augmentation de leur résistance, ceci laisse donc supposer qu’il s’agit dans la plupart du temps, d’infections à germes communautaires. La majorité des souches isolées présentait un profil de sensibilité aux antibiotiques correspondant à des souches sauvages, 85% des staphylocoques étaient sensibles à la méticilline, un seul pseudomonas était résistant à la ceftazidime, et il n’a pas été isolé de souches multi-résistantes même parmi les bactéries Gram négatifs.

En fonction du réservoir habituel des différentes espèces bactériennes isolées on peut supposer que la contamination se fait essentiellement à partir de la flore environnementale (40%), et cutanée (35%) plutôt que de la flore endogène (25%).

Trois cathéters (15%) ont été retirés définitivement avec transfert secondaire des patients en hémodialyse. Ce nombre est inférieur à celui rapporté par Narayan et al. en Inde (29%) [16], ou par Gadola et al. (22%) en Uruguay [17].

Seize pourcent des décès en DPCA à Hong Kong [21] et 18% aux Etats Unis [22] sont liés aux PI. Dans notre centre de DP, Aucun décès suite à une PI n’est survenu.

## Conclusion

La PI est une complication grave et redoutable de la DP. Le taux de PI dans notre centre de DPCA est de 21m-p ce qui correspond au taux acceptable définie par les sociétés internationales. Les germes les plus responsables de ces infections dans notre centre sont les bactéries à Gram négatif, et la contamination semble être manuportée se faisant essentiellement à partir de la flore environnementale, et cutanée plutôt que de la flore endogène. Les avancées techniques apportées à la DP notamment l’introduction du système double poche, ainsi que les efforts de recyclage et de formation du personnel soignant et des patients eux-mêmes, devraient aboutir à la diminution des péritonites impliquant les germes cutanés et environnementaux notamment *Staphylocoque aureus*.
